# Vehicle-to-everything decision optimization and cloud control based on deep reinforcement learning

**DOI:** 10.1038/s41598-025-12772-3

**Published:** 2025-08-09

**Authors:** Zhenhai Gao, Dayu Liu, Chengyuan Zheng

**Affiliations:** 1 College of Automotive Engineering and the National Key Laboratory of Automotive Chassis Integration and Bionics, Jilin University, Changchun, 130025 China; 2 College of Automotive Engineering, Jilin University, Changchun, 130025 China

**Keywords:** Autonomous driving, Vehicle-to-everything, Deep reinforcement learning, Decision optimization, Hazard classification, Engineering, Materials science

## Abstract

To address the challenges of decision optimization and road segment hazard assessment within complex traffic environments, and to enhance the safety and responsiveness of autonomous driving, a Vehicle-to-Everything (V2X) decision framework is proposed. This framework is structured into three modules: vehicle perception, decision-making, and execution. The vehicle perception module integrates sensor fusion techniques to capture real-time environmental data, employing deep neural networks to extract essential information. In the decision-making module, deep reinforcement learning algorithms are applied to optimize decision processes by maximizing expected rewards. Meanwhile, the road segment hazard classification module, utilizing both historical traffic data and real-time perception information, adopts a hazard evaluation model to classify road conditions automatically, providing real-time feedback to guide vehicle decision-making. Furthermore, an autonomous driving cloud control platform is designed, augmenting decision-making capabilities through centralized computing resources, enabling large-scale data analysis, and facilitating collaborative optimization. Experimental evaluations conducted within simulation environments and utilizing the KITTI dataset demonstrate that the proposed V2X decision optimization method substantially outperforms conventional decision algorithms. Vehicle decision accuracy increased by 9.0%, rising from 89.2 to 98.2%. Additionally, the response time of the cloud control system decreased from 178 ms to 127 ms, marking a reduction of 28.7%, which significantly enhances decision efficiency and real-time performance. The introduction of the road segment hazard classification model also results in a hazard assessment accuracy of 99.5%, maintaining over 95% accuracy even in high-density traffic and complex road conditions, thus illustrating strong adaptability. The results highlight the effectiveness of the proposed V2X decision optimization framework and cloud control platform in enhancing the decision quality and safety of autonomous driving systems.

**Supplementary Information:**

The online version contains supplementary material available at 10.1038/s41598-025-12772-3.

## Introduction

The rapid advancement of autonomous driving technology is driving significant changes in the transportation sector. At its core, autonomous driving seeks to improve traffic safety, reduce accidents, and optimize traffic efficiency through intelligent decision-making systems^1–3^. However, as traffic environments grow increasingly complex, autonomous driving systems encounter challenges in navigating intricate road conditions, fluctuating traffic flows, and unpredictable events^4,5^. The ability to make accurate and timely vehicle decisions in such environments, ensuring both safety and responsiveness, has emerged as a critical concern in autonomous driving research^6,7^.

Presently, most autonomous driving systems rely on on-board perception and decision-making modules to process sensory data and determine vehicle behaviour^8–10^. This single-vehicle intelligence model, however, faces limitations in handling complex traffic situations, as it depends solely on the vehicle’s immediate sensory inputs. The lack of comprehensive data sources often hinders the system’s ability to make precise and timely decisions under dynamic conditions. In response, Vehicle-to-Everything (V2X) technology has surfaced as a promising solution to enhance the decision-making capabilities of autonomous driving systems^11,12^. By linking vehicles, traffic infrastructure, and cloud platforms, V2X enables real-time data exchange, allowing for more accurate decision-making and the execution of actions^13,14^. This collaborative approach not only improves decision efficiency in dynamic traffic environments but also enhances the adaptability and safety of autonomous driving systems.

Despite the considerable theoretical advantages offered by V2X technology, a significant technical challenge in its practical application persists: efficiently integrating information from various sensors and traffic infrastructure, and utilizing intelligent algorithms to enable rapid and accurate decision-making^15–17^. Traditional decision-making methods in autonomous driving, primarily reliant on rule-based or model-based algorithms, struggle to perform effectively in complex traffic environments^18^. In contrast, deep reinforcement learning (DRL), a sophisticated approach that optimizes decision-making through a trial-and-error process, has emerged as a pivotal technology in autonomous driving^19^. DRL enables systems to autonomously learn and continuously refine decision strategies, facilitating more efficient decision-making in dynamic, complex environments^20–22^.

Road segment hazard assessment plays a crucial role in autonomous driving systems, serving as an essential element in ensuring safety and optimal performance. By continuously evaluating the hazard levels of road segments in real time, the system can detect and avoid high-risk areas, adapting driving strategies to reduce the likelihood of accidents^23^. Traditional hazard assessment methods, which typically rely on historical data or simple rule-based systems, fall short when faced with the complexities and dynamic nature of traffic conditions. As a result, the integration of deep learning techniques, combining historical data with real-time perception inputs, has emerged as a critical strategy for intelligent road hazard classification, significantly enhancing the decision-making capabilities of these systems.

To advance the decision-making efficiency of autonomous driving systems, this research proposes a three-tier collaborative V2X decision-making architecture. This architecture integrates Hierarchical Reinforcement Learning (HRL) with multi-agent collaborative optimization to establish a highly efficient and intelligent autonomous driving decision framework. The model employs a division of labor among vehicle-side agents, roadside agents, and cloud-side agents, covering near-field perception, regional situational assessment, and global optimization, respectively. Each agent undertakes tasks corresponding to different perception ranges and decision-making temporalities. By incorporating an Option-Critic structure within the HRL framework, the system achieves hierarchical abstraction of the action space, facilitating the collaborative execution of multi-level policies. The multi-agent framework enables each agent to maintain a dynamic equilibrium between local optimality and global objectives, thereby accelerating policy convergence and enhancing decision robustness. A risk classification model, based on a convolutional neural network (CNN), fuses multi-source sensor data to achieve multi-level risk assessment. Furthermore, an autonomous driving cloud control platform is introduced. This platform leverages distributed computing resources and big data analytics to achieve collaborative optimization, ultimately improving the accuracy and responsiveness of vehicle decisions.

## Literature review

Autonomous driving decision-making presents a central challenge in the pursuit of intelligent driving systems. Traditional methods of decision-making are often grounded in rule-based or model-based algorithms^24^. Early systems in autonomous driving largely depended on preset rules or optimization strategies based on the Markov decision process. The introduction of the deep Q-network by Lu et al.^25^ marked a significant advancement, opening new possibilities for integrating reinforcement learning into autonomous driving. By incorporating deep neural networks, this method addresses the curse of dimensionality in the Q-learning algorithm, leading to notable improvements in decision-making performance. Despite these advancements, the algorithm continues to face limitations, particularly in adapting to the dynamic changes inherent in complex traffic environments, especially regarding exploration efficiency and decision-making speed. In response, recent research has focused on refining DRL algorithms to enhance both the quality and efficiency of decisions.

For example, Yuan et al.^26^ introduced a DRL-based framework for autonomous driving that dynamically adjusts decision strategies through continuous interaction with the environment. This approach demonstrated high decision accuracy across various driving scenarios. In parallel, Nugroho et al.^27^ applied policy gradient algorithms, showing through simulation experiments the effectiveness of DRL-based strategies in high-density traffic settings. However, these studies predominantly concentrate on optimizing individual decision modules for vehicles, often neglecting the broader scope of V2X systems.

V2X technology serves as a fundamental enabler for enhancing the efficiency and safety of autonomous driving systems by facilitating real-time information exchange. In recent years, research on V2X technology has expanded significantly, contributing to its increasing integration into autonomous driving frameworks. He et al.^28^ introduced a V2X-based cooperative framework that utilizes real-time data exchange between vehicles and road infrastructure to dynamically adjust traffic signals and driving strategies, optimizing overall traffic flow. Bin and Sun^29^ examined cooperative decision-making within V2X systems and proposed a dynamic information-sharing model between vehicles and road infrastructure, leading to notable improvements in traffic safety and driving stability. While these studies underscore the potential of V2X in autonomous driving, most existing implementations primarily focus on the design and deployment of information exchange infrastructure, with limited integration into intelligent decision-making processes. A key challenge remains in effectively utilizing real-time data from both vehicles and road infrastructure to enhance decision optimization, enabling autonomous systems to respond adaptively to dynamic traffic conditions.

In recent years, deep learning methods have been extensively applied to road risk assessment, demonstrating substantial advancements in predictive accuracy. Feng et al.^30^ introduced a risk assessment model utilizing a CNN to process real-time perception data, enabling road risk prediction to support autonomous driving decisions. However, reliance on a single data source remains a limitation, as such methods often lack the integration of multimodal data necessary for a more comprehensive risk evaluation. Addressing this challenge, risk assessment frameworks incorporating multimodal data fusion have garnered increasing attention. Zhao et al.^31^ proposed a joint model that integrates vehicle sensor data with historical traffic records to assess road segment risk, significantly enhancing evaluation accuracy.

Cloud computing platforms have become integral to autonomous driving applications, offering large-scale data analysis and collaborative optimization capabilities. While existing cloud-based control platforms are widely adopted, they primarily focus on information transmission and traffic signal scheduling. This limits their support for real-time decision optimization, particularly lacking deep policy collaboration mechanisms between vehicle-level and roadside intelligent agents. Furthermore, current research integrating CNN for risk prediction often employs centralized perception data processing, failing to establish a direct coupling between risk assessment and hierarchical decision-making. In response to these challenges, this research proposes a three-tier V2X decision framework based on HRL and multi-agent collaboration. This framework integrates a CNN-based road segment risk classification model and a cloud-based distributed control platform.

Compared to existing methods, the proposed framework offers significant advantages in the following aspects:

1. Structural Innovation: By mapping the reinforcement learning policy structure to a “vehicle-road-cloud” three-tier intelligent agent architecture, this approach overcomes the limitations of traditional methods that decouple perception and decision-making.
2. Methodological Advancement: This research introduces an integrated method combining the Option-Critic structure with multi-agent joint training, achieving integrated optimization of cross-agent hierarchical policies.
3. Practical Application: The framework establishes a closed-loop linkage mechanism between risk perception and policy control by driving high-level intention planning with CNN risk assessment results.
4. This method significantly enhances the system’s dynamic response capability and global optimization performance in complex traffic environments, expanding a new mode of decision collaboration for V2X architectures in intelligent driving scenarios.

## Research methodology

### Hierarchical collaborative V2X decision architecture design

The V2X decision-making framework plays a pivotal role in autonomous driving systems by leveraging vehicle-to-infrastructure collaboration to enhance decision intelligence and precision. Traditional autonomous driving frameworks rely exclusively on onboard perception and computational resources, limiting the scope of environmental awareness and adaptability. In contrast, the V2X decision-making framework incorporates roadside units (RSUs), transportation infrastructure, and cloud computing platforms, facilitating decision-making informed by a broader range of environmental data while enabling dynamic collaborative optimization. Building upon these insights, this research introduces a three-tier collaborative V2X decision framework. This framework comprises three distinct layers: the Local Agent (vehicle-side agent), the Edge Agent (roadside agent), and the Global Agent (cloud-side agent). Each agent is designed to collaborate through a division of labor concerning its state perception range, decision-making timeliness, and assigned weight, significantly enhancing the system’s response speed and global optimization capabilities.

Several key components constitute this framework.

1. Local Agent: Situated directly within the vehicle, the Local Agent acquires real-time data from on-board sensors such as LiDAR, cameras, and millimeter-wave radar. It leverages local neural network models to rapidly perceive surrounding static and dynamic obstacles, lane lines, and pedestrians. The Local Agent holds the highest response priority for time-sensitive, near-field operations like emergency braking and lane keeping, and possesses a degree of autonomous planning capability.
2. Edge Agent: Deployed at RSUs or traffic infrastructure nodes, the Edge Agent is responsible for aggregating multi-vehicle perception information and traffic signal status within its local road network. This includes data such as signal phases, construction information, and traffic flow density. Through edge computing, the Edge Agent achieves regional situational awareness and dispatches collaborative recommendations to Local Agents within its area, such as speed limits, signal timing responses, or temporary path adjustments, thereby improving local traffic efficiency.
3. Global Agent: Functioning as the central hub for global optimization, the Global Agent is deployed on a cloud control platform. It constructs a large-scale Traffic State Map by integrating historical traffic data, road condition predictions, and real-time perceptual data. It then executes decision computations based on deep reinforcement learning and global optimization algorithms. The Global Agent formulates medium-to-long-term driving strategies, such as path replanning and high-risk area avoidance, and relays these strategies to the Edge and Local layers in a low-frequency, high-value manner.

During system operation, the Local Agent, Edge Agent, and Global Agent achieve real-time information synchronization and policy collaboration through a high-bandwidth, low-latency V2X communication network. This three-tier agent architecture demonstrates a clear hierarchical collaborative relationship in terms of perception range, decision-making timeliness, and feedback weighting. In terms of perception range, the Local Agent primarily relies on in-vehicle sensors, giving it a relatively small perception radius. It focuses on processing the near-field dynamic environment within approximately 50 m, including nearby vehicles, pedestrians, and obstacles. The Edge Agent, by contrast, acquires broader traffic information through RSUs deployed at critical road nodes. This allows it to cover localized traffic areas such as intersections and ramps. The Global Agent integrates data from multiple regions to construct a city-level global traffic state map, enabling comprehensive perception and prediction of wide-area traffic environments. At the decision-making response level, different agents assume distinct decision-making roles based on their perceptual capabilities and information granularity. The Local Agent executes millisecond-level emergency decisions with the fastest response speed, such as obstacle avoidance, lane keeping, and yielding strategies. These are suited for highly dynamic and temporary scenarios. The Edge Agent is responsible for regional traffic coordination, performing medium-frequency, medium-response level operations like traffic flow guidance, signal priority strategies, and temporary road controls. The Global Agent focuses on global path planning and policy optimization. It formulates medium-to-long-term driving strategies, such as path replanning and congestion avoidance, based on historical data and predictive models. While its response frequency is relatively lower, its decision depth and global weighting are the highest.

To effectively navigate diverse traffic environments and network conditions, this system incorporates a dynamic feedback weighting mechanism. This mechanism intelligently adjusts the dominance of the three agent types in decision-making. When communication latency increases or a local network experiences an outage, the system automatically elevates the Local Agent’s decision priority. This ensures the vehicle’s fundamental operational safety. In the event of a sudden incident in a specific area, such as an accident or temporary road closure, the Edge Agent leverages its local contextual advantage to rapidly generate and issue emergency dispatch commands. During normal operations and stable traffic conditions, the Global Agent takes the lead in overall decision optimization, providing proactive strategic support based on a global traffic model. Through this multi-level, dynamically adjustable collaborative mechanism, the V2X decision framework can balance real-time responsiveness, local coordination, and global optimality. This enables efficient and robust autonomous driving decision support in complex traffic environments.

**Fig. 1:**
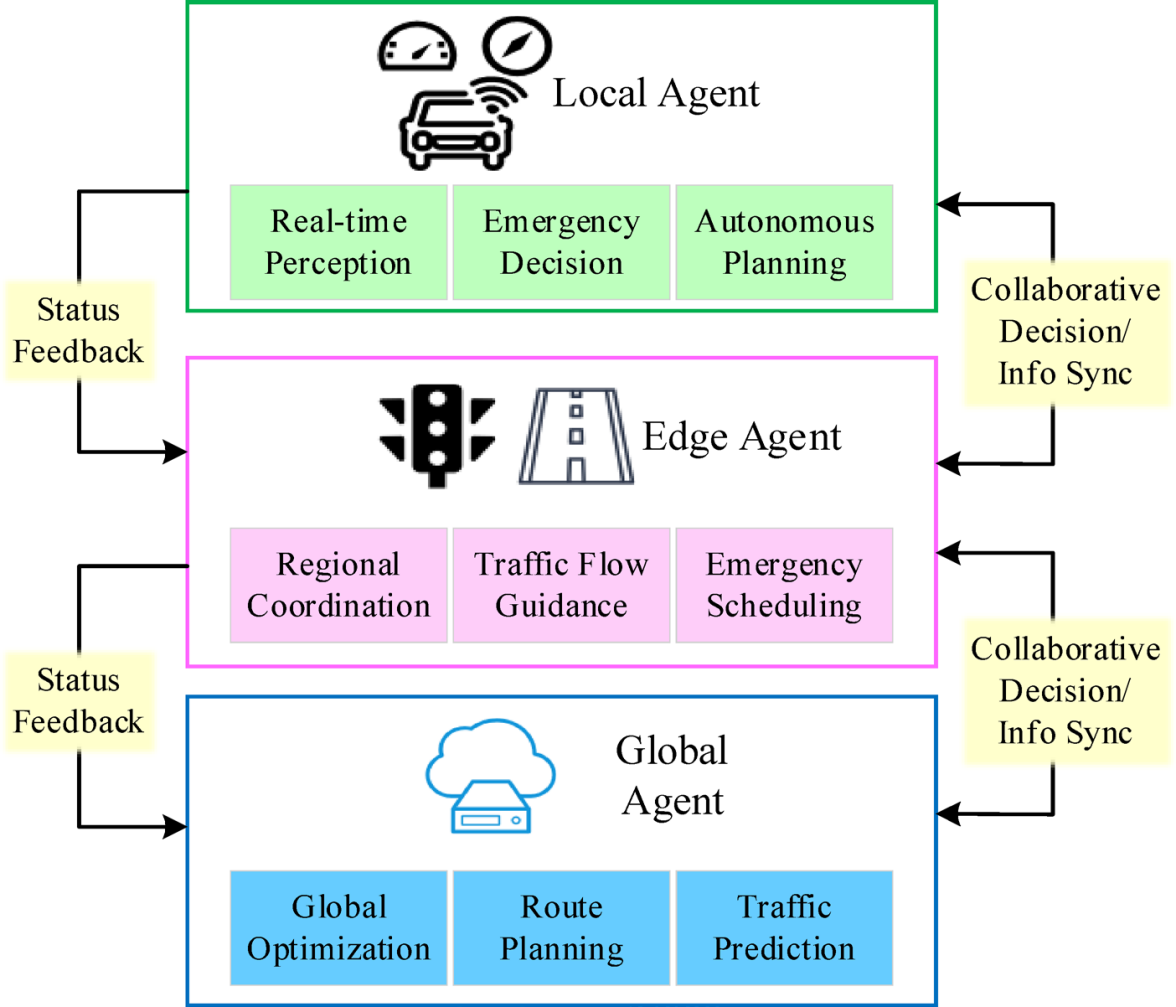
Three-tier collaborative V2X decision framework.

The execution module translates the integrated results of the three-tier decisions into concrete vehicle operations, including acceleration, braking, and steering control. Simultaneously, the vehicle continuously feeds real-time status information—such as speed, acceleration, and obstacle data—back to the Edge and Global Agents, forming a multi-level closed-loop feedback mechanism. This enables the cloud platform to maintain macroscopic control while leveraging the rapid response capabilities of the Edge and Local Agents, achieving dynamic adaptation and precise control in complex traffic scenarios. Figure 1 depicts this three-tier collaborative V2X decision framework.

### HRL and multi-agent collaborative optimization

Within an autonomous driving system, the decision-making module serves as the mechanism through which vehicle perception data is translated into specific driving actions, such as acceleration, braking, and steering. To adapt to complex, dynamic, and time-varying traffic environments, this research enhances traditional DRL by incorporating HRL and Multi-Agent Reinforcement Learning (MARL) mechanisms. This approach enables hierarchical policy modeling and multi-agent perception collaboration. By introducing intermediate decision granularities called “options,” the raw action space is abstracted into higher-level policy units. This creates a unified mapping within the three-tier V2X collaborative structure:

1. The Cloud Agent corresponds to the High-level Policy.
2. The Edge Agent corresponds to the Option Policy.
3. The Local Agent executes the Low-level Policy.

In formal modeling, the reinforcement learning task is defined as a Markov Decision Process (MDP), comprising state space (*S*), action space (*A*), reward function ($\:R:S\times\:A\to\:\mathbb{R}$), and policy function ($\:\pi\::S\to\:A$). Here, the state space (*S*) includes the vehicle’s own states (e.g., position, velocity, acceleration), environmental states (e.g., neighboring vehicles, traffic light status, obstacles), and V2X perception information such as vehicle-to-vehicle and vehicle-to-road communication states. The action space (*A*) at the low level encompasses continuous actions like acceleration, deceleration, and steering, while at the high level, it is abstracted into policy options such as “requesting passage,” “waiting,” or “lane changing.” The reward function *R* is comprehensively designed by considering factors like driving safety, traffic efficiency, and comfort. The state transition mechanism is jointly determined by the behavior of traffic participants, changes in traffic management signals, communication delays, and external disturbances, reflecting the dynamism and uncertainty of real traffic systems.

The objective is to learn an optimal policy $\:{\pi\:}^{*}$ that maximizes the expected cumulative discounted reward $\:J\left(\pi\:\right)$, defined as follows:

1 $$\:J\left(\pi\:\right)={\mathbb{E}}_{\pi\:}\left[\sum\limits_{t=0}^{\infty\:}{\gamma\:}^{t}R({s}_{t},{a}_{t})\right]$$

In the HRL framework, the policy function is further decomposed into a high-level policy $\:{\pi\:}_{H}\left(o\right|s)$, a low − level policy $\:{\pi\:}_{L}\left(a\right|s,o)$, and a termination condition $\:{\beta\:}_{o}\left(s\right)\in\:\left[\text{0,1}\right]$. This triplet $langle {\pi _{L} ,\beta ,\pi _{H} } \rangle$ forms the Option-Critic architecture, which enables the agent to autonomously select an appropriate option (i.e., a policy unit) based on the current state and to dynamically determine whether to terminate the current option and re-evaluate the high-level policy during execution. Within the Option-Critic framework, the updates of both the high-level policy $\:{\pi\:}_{L}$ and the low-level action policy $\:{\pi\:}_{L}$ rely on policy gradient methods, optimizing the objective of maximizing the expected cumulative reward. Specifically, the parameterized high-level policy is denoted as $\:{\pi\:}_{H}\left(o\right|s;{\theta\:}_{H})$, the low − level policy as $\:{\pi\:}_{L}\left(a\right|s,o;{\theta\:}_{L})$, and the termination function $\:{\beta\:}_{o}\left(s;{\theta\:}_{\beta\:}\right)$ represents the probability of terminating option *o* in state *s*. All components are modeled using differentiable parameters *θ*. The policy gradient for the high-level policy is defined in terms of the option-value function $\:{Q}_{{\Omega\:}}(s,o)$ and the state-value function $\:{V}_{{\Omega\:}}\left(s\right)$ as follows:

2 $$\:{\nabla\:}_{{\theta\:}_{H}}J\left({\theta\:}_{H}\right)=\mathbb{E}\left[{\nabla\:}_{{\theta\:}_{H}}\text{log}{\pi\:}_{H}\left(o\right|s;{\theta\:}_{H}\left)\right({Q}_{{\Omega\:}}\left(s,o\right)-{V}_{{\Omega\:}}\left(s\right))\right]$$

In Eq. (2), $\:{Q}_{{\Omega\:}}(s,o)$ denotes the expected cumulative discounted reward obtained by selecting option o in state *s*, and the state-value function $\:{V}_{{\Omega\:}}\left(s\right)$ is given by Eq. (3):

3 $$\:{V}_{{\Omega\:}}\left(s\right)=\sum\limits_{o}{\pi\:}_{H}\left(o\right|s\left){Q}_{{\Omega\:}}\right(s,o)$$

The low-level action policy π_L is also updated using the policy gradient method, with the gradient expressed as Eq. (4):

4 $$\:{\nabla\:}_{{\theta\:}_{L}}J\left({\theta\:}_{L}\right)=\mathbb{E}\left[{\nabla\:}_{{\theta\:}_{L}}\text{log}{\pi\:}_{L}\left(a\right|s,o;{\theta\:}_{L}\left)A\right(s,o,a)\right]$$

In Eq. (4), $\:A(s,o,a)$ is the advantage function defined as Eq. (5):

5 $$\:A\left(s,o,a\right)=Q(s,o,a)-{Q}_{{\Omega\:}}(s,o)$$

The advantage function measures the relative benefit of a specific action *a* compared to the overall value of the selected option *o*. The termination function $\:{\beta\:}_{o}\left(s;{\theta\:}_{\beta\:}\right)$ dynamically determines whether the current option should be terminated, thereby triggering the high-level policy to reselect a new option. Its gradient update direction is guided by the difference in expected return after option termination:

6 $$\:{\nabla\:}_{{\theta\:}_{\beta\:}}J\left({\theta\:}_{\beta\:}\right)=\mathbb{E}\left[{\nabla\:}_{{\theta\:}_{\beta\:}}{\beta\:}_{o}\left(s;{\theta\:}_{\beta\:}\right)\right({Q}_{{\Omega\:}}\left(s,o\right)-{V}_{{\Omega\:}}\left(s\right)-\eta\:\left)\right]$$

In Eq. (6), $\:\eta\:$ is a termination penalty term designed to prevent excessively frequent terminations. The termination function is typically parameterized using a sigmoid function to ensure that the output lies within the probability range [0, 1].

To enhance the coordination capability of the system, this mechanism is further extended to a multi-agent setting. In the three-layer V2X system architecture, multiple agents—namely vehicles, RSUs), and the cloud platform—act as independent agents making cooperative decisions. The overall system objective is formulated as the maximization of the joint expected return:

7 $$\:J\left({\pi\:}_{1},\cdots\:,{\pi\:}_{n}\right)={\mathbb{E}}_{{\pi\:}_{1},\cdots\:,{\pi\:}_{n}}\left[\sum\limits_{t=0}^{\infty\:}{\gamma\:}^{t}\sum\limits_{i=1}^{n}{R}_{i}({s}_{t}^{i},{a}_{t}^{i})\right]$$

In Eq. (7), *n* denotes the number of agents, and $\:{R}_{i}$ is the individual reward function for agent *i*.

For the joint policy optimization in the multi-agent setting of the three-layer V2X system, a Centralized Training with Decentralized Execution framework is adopted. During the centralized training phase, the state, action, and reward information from all agents are evaluated by a unified Critic network. The Critic takes the joint state-action pair $\:\left(s,a\right)$ as input and outputs the joint Q-value function:

8 $$\:{Q}_{tot}(s,a;{\theta\:}_{c})\approx\:\sum\limits_{i=1}^{n}{Q}_{i}({s}_{i},{a}_{i};{\theta\:}_{i})$$

Here:

9 $$\:s=({s}_{1},{s}_{2},\cdots\:,{s}_{n})$$

10 $$\:a=({a}_{1},{a}_{2},\cdots\:,{a}_{n})$$

In these expressions, $\:{\theta\:}_{c}$ denotes the parameters of the Critic, and $\:{\theta\:}_{i}$ represents the policy parameters of agent *i*. The joint Q-function serves as a global evaluation metric to guide the update of each agent’s local policy. In the decentralized execution phase, each agent independently selects its action based solely on its local observation $\:{s}_{i}$ and policy $\:{\pi\:}_{i}\left({a}_{i}\right|{s}_{i};{\theta\:}_{i})$, enabling online real-time decision-making while ensuring system scalability and responsiveness.

The multi-agent cooperation mechanism promotes global optimality by sharing partial environmental information and designing reward functions that incentivize coordination, thereby avoiding suboptimal behavior resulting from selfish local policies. The policy parameters are updated using a joint Q-value–based policy gradient:

11 $$\:{\nabla\:}_{{\theta\:}_{i}}J\left({\theta\:}_{i}\right)=\mathbb{E}\left[{\nabla\:}_{{\theta\:}_{i}}\text{log}{\pi\:}_{i}\left({a}_{i}\right|{s}_{i};{\theta\:}_{i}){Q}_{tot}\right(s,a;{\theta\:}_{c}\left)\right]$$

Moreover, communication protocols and coordination mechanisms are introduced to enable agents to achieve collaborative perception and policy adaptation through state sharing and policy exchange, further enhancing decision consistency and system robustness.

**Fig. 2:**
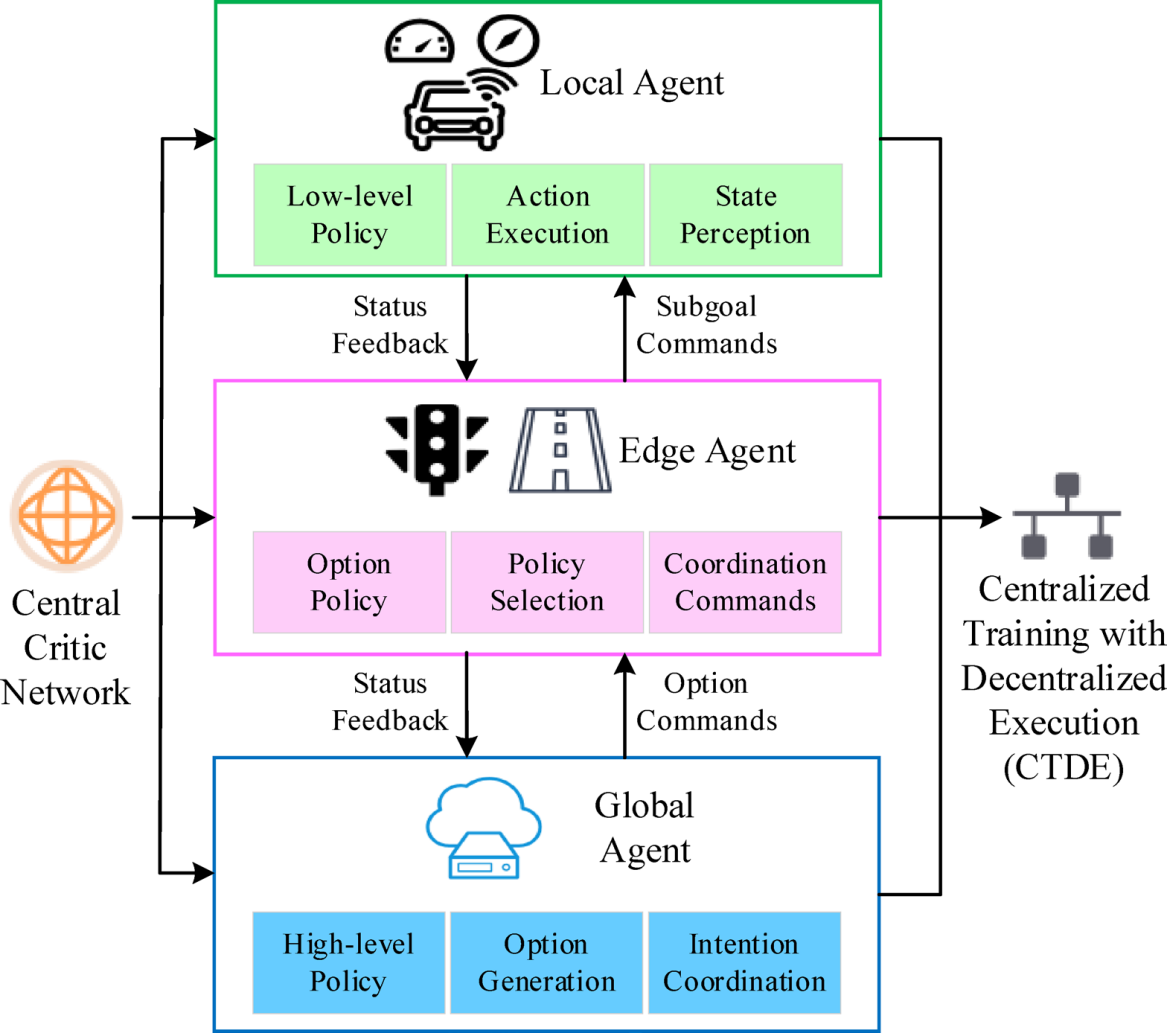
HRL-MARL architecture based on option-critic.

By incorporating a joint policy optimization mechanism, the MARL framework ensures that each agent dynamically balances individual optimality with the system-wide objective, avoiding convergence to locally suboptimal policies. The deployment architecture of the HRL-MARL framework based on the Option-Critic structure in autonomous driving systems is illustrated in Fig. 2.

As illustrated in Fig. 2, the HRL-MARL architecture, which uses the Option-Critic framework, assigns distinct roles to its three layers of agents: state perception, policy selection, and execution feedback. A Critic network evaluates the value of all policy layers, using the Q-function as a unified feedback signal. This guides effective collaboration between high-level policy intentions and low-level actions. Within a city-wide V2X network, multiple intelligent agents can operate simultaneously and influence each other. To ensure policy consistency, the system adopts a centralized training and distributed execution mechanism. This approach is particularly well-suited for urban and regional intelligent transportation environments that have robust V2X communication infrastructure and support multi-agent collaboration. Think of scenarios like smart intersections, multi-vehicle platoon coordination, and dynamic traffic management. This method does more than just improve policy convergence efficiency and stability. It significantly enhances the system’s responsiveness to sudden traffic events and complex interactive scenarios. This makes it especially valuable for autonomous driving tasks such as multi-vehicle coordination, intersection scheduling, and path negotiation.

### Road segment risk classification and autonomous driving cloud control platform design

**Fig. 3:**
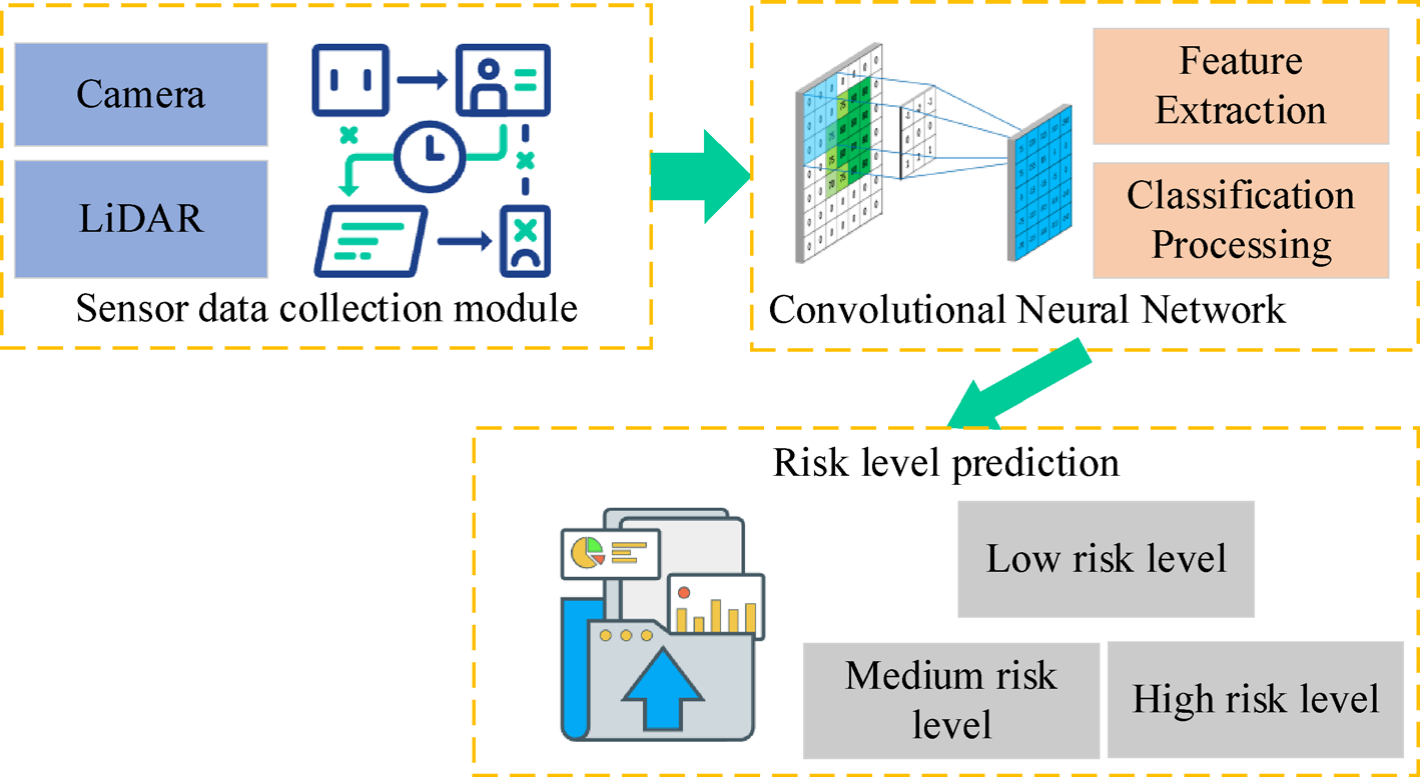
Road segment risk classification model.

To accurately assess road segment risks, a deep learning-based risk classification model is employed, leveraging CNNS to extract critical features from both real-time environmental data and historical sensor data. The model classifies and predicts risk levels, thus enhancing decision-making processes within autonomous driving systems. Figure 3 presents the architecture of the road segment risk classification model.

As illustrated in Fig. 3, sensor data from cameras, LiDAR, and radar sensors provide extensive information regarding the road and traffic environment. Image data undergo feature extraction via convolutional neural networks, which process the raw data to generate a risk score. The model architecture integrates multiple convolutional and pooling layers to capture spatial features, followed by classification through fully connected layers. The output is a risk level categorization, which includes three classes: low, moderate, and high risk. The risk classification model comprehensively considers the following key factors:

1. Traffic Participant Density: This includes the number of vehicles and pedestrians per unit area.
2. Speed Volatility: This encompasses the variance in speed of the ego vehicle and neighboring vehicles.
3. Spatial Constraint: This refers to structural risks such as lane width and distance to obstacles.
4. Historical Risk Labels: This corresponds to the accident frequency and historical violation records for a given road segment.

Based on these factors, risk levels are categorized as follows:

1. High-risk levels correspond to areas with high density of dynamic targets, frequent sudden speed changes, and spatially constrained regions.
2. Medium-risk areas are typically associated with moderate traffic density and occasional anomalous behaviors.
3. Low-risk areas refer to road scenarios with unobstructed structures, sparse participants, and stable behavior.

Training of the model utilizes historical data, incorporating accident records and road condition information from actual traffic situations, thereby refining its predictive capabilities. This risk classification model is deployed distributively within the three-tier collaborative architecture:

1. The Local Agent is responsible for the immediate identification of near-field risks.
2. The Edge Agent focuses on regional-level risk assessment for key road segments.
3. The Global Agent performs macroscopic-level global risk analysis.

The information fusion and collaborative inference among these three intelligent agents significantly enhance the timeliness and spatial coverage accuracy of risk identification, providing reliable support for upper-level driving decision strategies.

**Fig. 4:**
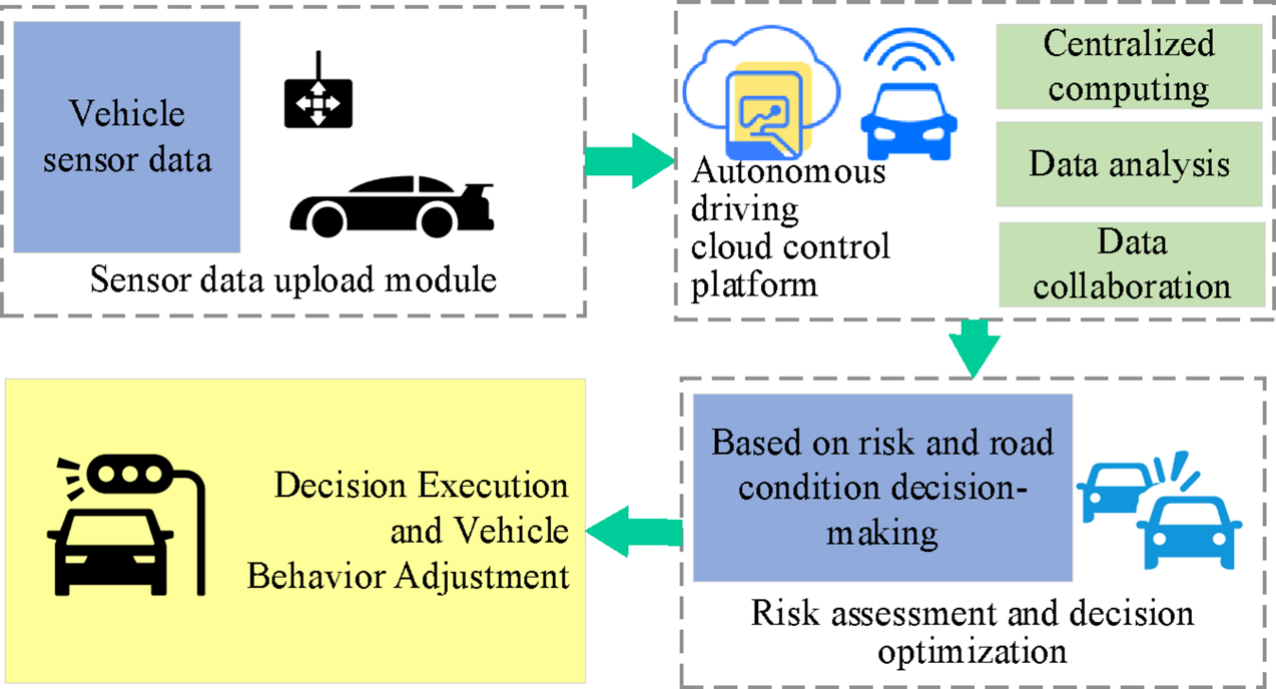
Autonomous driving cloud control platform architecture.

To enhance collaborative decision-making capabilities and response efficiency, an autonomous driving cloud control platform is implemented, as depicted in Fig. 4. This platform employs a distributed computing architecture, offering robust data processing and analytical capabilities. It facilitates real-time monitoring of vehicle statuses and generates comprehensive decisions based on sensor data collected from each vehicle. By utilizing cloud computing resources, the platform processes real-time data from all vehicles, delivering tailored driving strategies for each individual unit.

In scenarios with multiple vehicles sharing the same road segment, the cloud control platform plays a crucial role. It comprehensively considers each vehicle’s real-time status and the overall traffic situation. Based on a collaborative control mechanism, it dynamically adjusts vehicle paths and speeds, effectively alleviating congestion, reducing collision risks, and enhancing traffic flow continuity and safety. High-speed, two-way data exchange between on-board units and the cloud platform ensures the real-time transmission of critical vehicle status parameters, such as speed, acceleration, steering angle, and obstacle information. This data supports the platform’s application of deep learning and reinforcement learning models for continuous optimization of decision-making strategies. Through iterative algorithm updates, the cloud platform consistently improves its ability to respond to sudden road hazards, obstacles, and traffic disruptions, achieving an intelligent evolution of vehicle autonomous decision-making. Furthermore, the platform possesses environmental context awareness. It can rapidly adjust decision plans based on multi-dimensional real-time data, including pedestrian dynamics, obstacle distribution, and surrounding vehicle behavior, ensuring the timeliness and accuracy of strategy responses. This dynamic adjustment mechanism is a core function of the platform, effectively enhancing system safety and adaptability in diverse driving scenarios. Cloud-based strategies not only improve the reaction speed and decision quality of individual vehicles but also strengthen overall driving safety through inter-vehicle information sharing and collaborative management. In the event of sudden incidents, such as emergency braking, the cloud platform can swiftly broadcast relevant information to surrounding vehicles, enabling them to adjust their driving status in a timely manner and reduce the probability of chain accidents.

In conclusion, the cloud control platform substantially elevates the safety, responsiveness, and performance of autonomous driving systems. By leveraging real-time data acquisition, collaborative decision-making, and dynamic, context-aware adjustments, the platform improves not only the decision-making efficiency of individual vehicles but also the broader coordination and risk management across the fleet. This ensures timely and precise decision-making in complex driving environments, ultimately enhancing both the performance of the autonomous system and the safety of the road network.

### Experimental setup and evaluation

The simulation environment utilized in the experiments integrates CarSim and SUMO, tools designed for vehicle dynamics modeling and traffic flow simulation, respectively. CarSim simulates vehicle behavior under diverse driving conditions, including acceleration, speed, and maneuverability, while SUMO provides a representation of the road network, traffic flow, and the behavior of surrounding vehicles, creating a realistic environment for testing. The combination of these tools facilitates the modeling of various driving scenarios and traffic conditions. This research establishes a robust data exchange between CarSim and SUMO via Traffic Control Interface (TraCI). This real-time synchronization of vehicle state information across both platforms enables the dynamic modeling and control response necessary for a complete “perception-decision-control” closed loop. CarSim’s realistic vehicle dynamics feedback is used to update vehicle states within SUMO, allowing the simulation of authentic vehicle behavior within a traffic scenario. To support collaborative decision-making among multiple intelligent agents, a V2X communication module is integrated into the experimental environment. This module simulates real-time information exchange between vehicle-side and roadside units, encompassing the sharing of perception data, broadcasting of risk levels, and transmission of collaborative control signals. Leveraging this V2X communication architecture, the three layers of intelligent agents—vehicle, roadside, and cloud—achieve synchronized state updates and collaboratively generate decisions.

To comprehensively evaluate the performance of the road risk classification model and the autonomous driving decision-making system, the KITTI dataset was employed. This dataset, containing real-world driving data such as traffic signs, road conditions, obstacle detection, and road condition changes, serves as a valuable resource for testing the V2X decision-making framework under complex road conditions. For the experiments, a subset of the KITTI dataset is selected, specifically including complex scenarios such as urban roads, intersections, and disturbances from pedestrians and non-motorized vehicles. This subset serves as the source for training and validating the perception data.

The raw data undergoes the following preprocessing steps:

1. Timestamp synchronization for image and LiDAR data occurs.
2. Spatial distribution features for traffic objects are extracted using annotation information.
3. Data augmentation (e.g., rotation, lighting variations) is applied to enhance model robustness.
4. Risk labeling is performed for each frame—categorizing scenes into high, medium, or low risk—based on accident records and scene context. This generates the labeled dataset for training the risk classification model.

The processed data is then integrated into the SUMO/CarSim environment to augment perception inputs and simulate realistic driving situations. By integrating the KITTI dataset with the simulation environment, a more thorough performance assessment of the framework is possible. Cross-validation was used in training and validating the models, ensuring the robustness of the system across various scenarios. Multiple rounds of experimentation were conducted, concentrating on the system’s adaptability in complex urban intersections marked by mixed traffic involving pedestrians, high vehicle density, and occlusions. These experiments aimed to evaluate the decision-making efficacy and stability of the system in high-risk contexts.

**Fig. 5:**
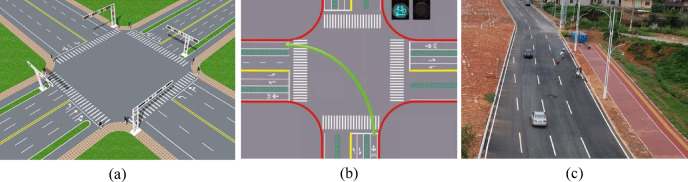
Schematic diagrams of different experimental scenarios (**a** Scenario 1; **b** Scenario 2; **c** Scenario 3).

The simulation results are generated through multiple experimental runs and specifically designed evaluation scenarios. For testing within urban intersection environments, various intersection types were selected, including signal-controlled intersections (Scenario 1), unprotected left-turn intersections (Scenario 2), and multi-lane merging zones (Scenario 3), to validate the system’s adaptability in high-density traffic situations. The schematic diagram of different scenarios is shown in Fig. 5. Scenario 1 is a crossroads where traffic signals control the driving rules of vehicles in different directions. The unprotected left turn intersection in Scenario 2 refers to the intersection not having a dedicated left turn signal, but sharing a traffic light with the straight ahead. The multi-lane merging area in Scenario 3 refers to the road section or intersection where two lanes merge into one lane.

**Fig. 6:**
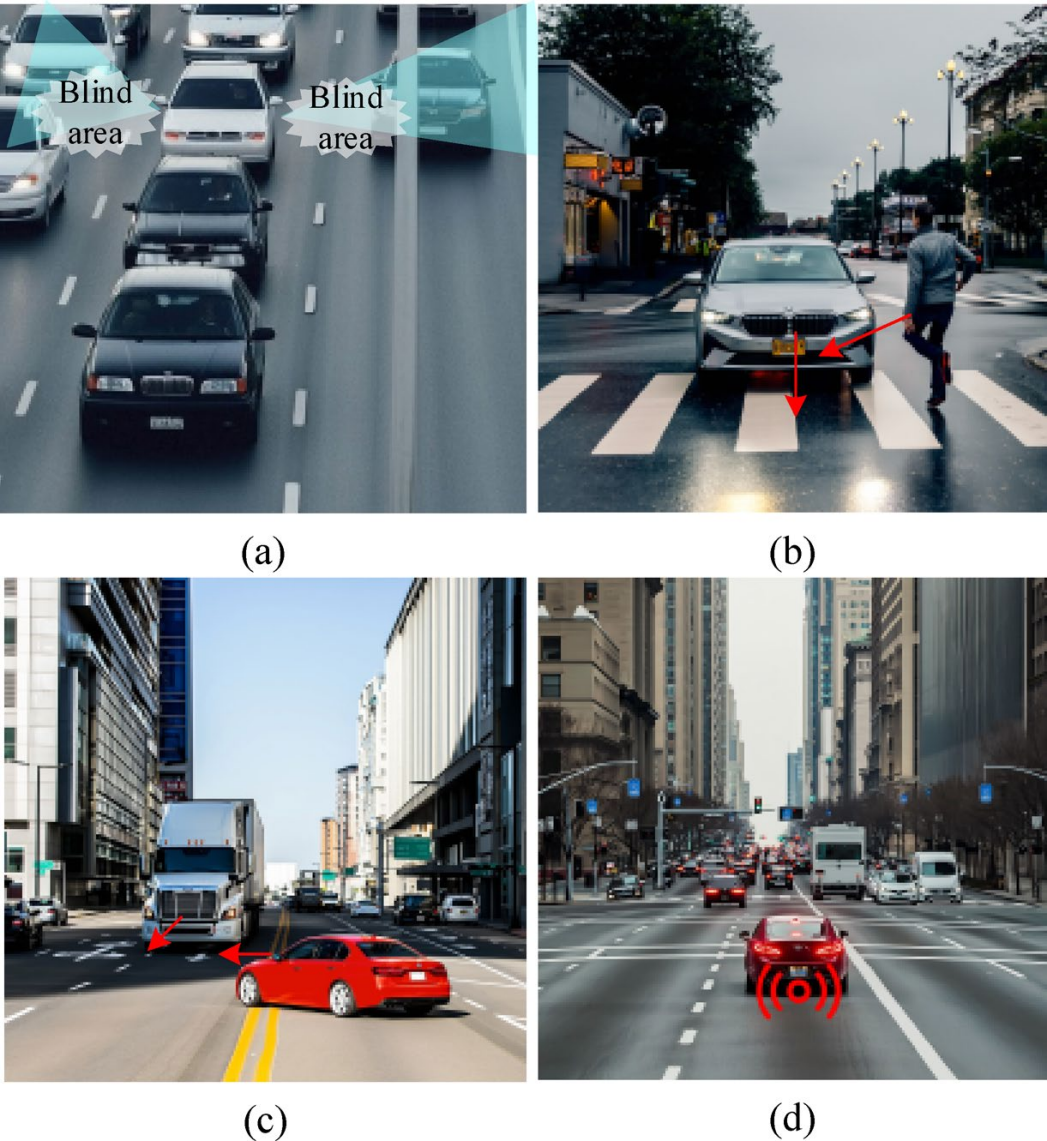
Schematic diagram of different emergency situations (a. Emergency Scenario 1; b. Emergency Scenario 2; c. Emergency Scenario 3; d. Emergency Scenario 4).

By operating the CarSim-SUMO co-simulation environment, key metrics for each experimental cycle are recorded and extracted. These metrics include vehicle behavior trajectories, decision latency, risk response, and system stability. To assess the system’s ability to respond to emergencies, four typical sudden incident scenarios are defined. Emergency scenarios included occlusion-induced perception blind spots (Emergency Scenario 1), pedestrians abruptly entering the road (Emergency Scenario 2), vehicles executing illegal lane changes (Emergency Scenario 3), and sudden halts by the leading vehicle (Emergency Scenario 4). The schematic diagram of different emergency scenarios is shown in Fig. 6. These scenarios simulated typical complex intersection conditions to evaluate the system’s responsiveness and decision-making performance during unanticipated events.

For each of the four typical sudden incident scenarios, 20 simulation runs were conducted. The proposed method’s decision accuracy and reliability in these emergent situations were then evaluated by comparing its performance against traditional benchmark algorithms. Evaluation metrics encompassed decision accuracy, response times, risk recognition proficiency, and overall stability when operating within these intricate urban settings.

## Results and discussion

### Model performance evaluation

**Fig. 7:**
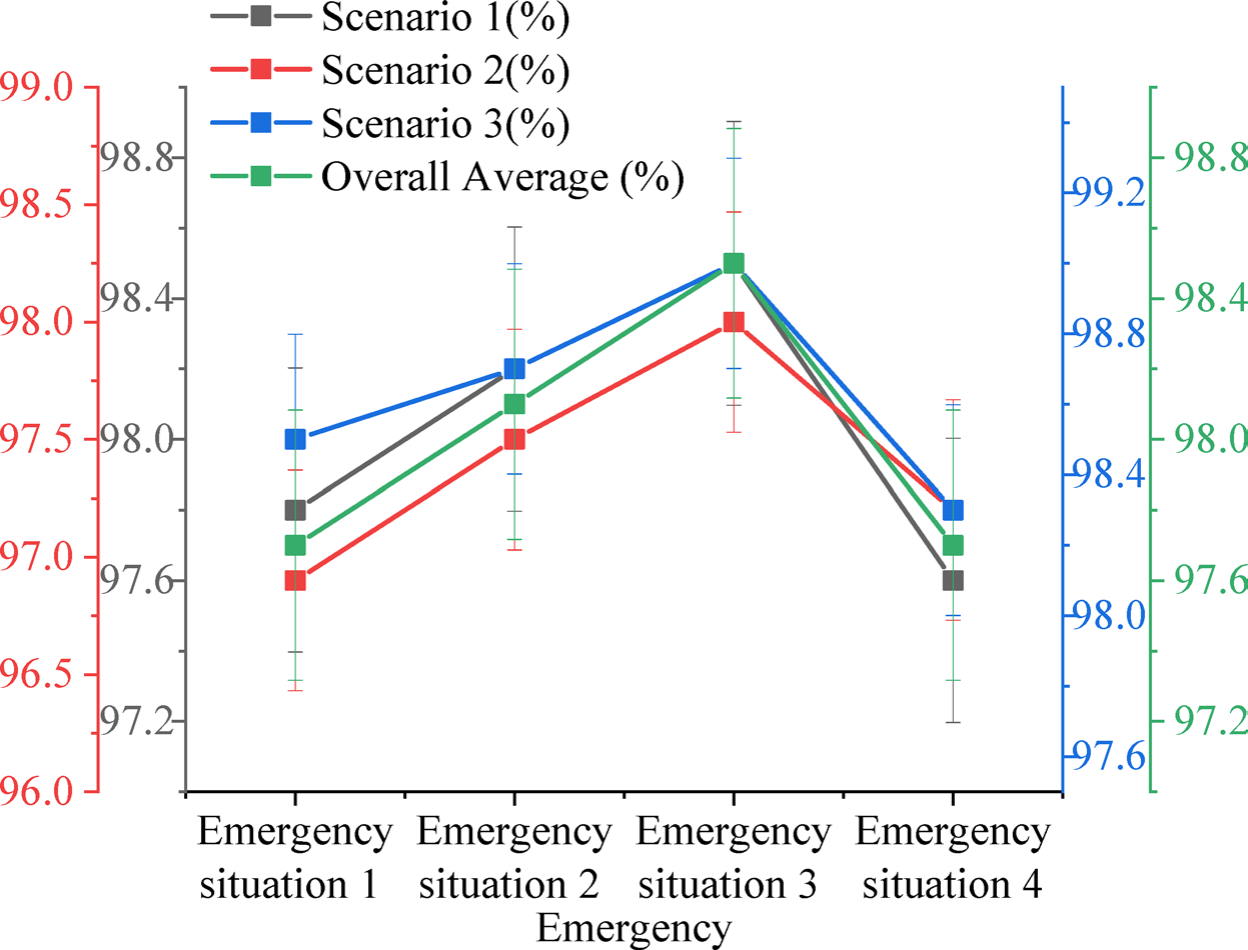
Decision accuracy under different emergency situations.

To ensure the applicability and stability of the proposed hierarchical collaborative V2X decision optimization system in a practical network deployment environment, performance tests were conducted under simulated real-world V2X communication network conditions. Specifically, the simulated environment incorporated various typical network constraint parameters, including communication latency (average delay of 30–50 milliseconds), bandwidth limitations (2 Mbps uplink, 5 Mbps downlink), and packet loss rates (up to 3%). These parameters were set based on actual test data from current 5G-V2X network and DSRC communication standards, aiming to maximize the fidelity of the simulated communication environment in vehicle-road cooperative networks. On this basis, the system’s response time and decision-making capabilities were comprehensively evaluated across various traffic emergency scenarios. This ensures that observed performance improvements were not merely achieved under idealized conditions but hold practical significance for real-world deployment. Furthermore, the testing platform adopts a distributed deployment architecture, where vehicle-side and roadside modules were connected to a cloud server via local simulated communication links, mimicking the communication structure of a city-scale V2X network. The impact of transmission characteristics under different network topologies on system response time was also included in the evaluation, thereby providing more practically valuable performance measurements. The experimental outcomes were scrutinized across various dimensions, including decision accuracy, response time, and risk assessment precision. Figure 7 illustrates the decision-making accuracy under varying emergency conditions at urban complex intersections.

Data presented in Fig. 7 highlight the system’s decision-making proficiency across different emergency scenarios within a complex urban intersection environment. The results indicate strong overall performance, particularly in adapting to high-density traffic and intricate road conditions. In scenarios involving perception blind spot occlusion (Emergency Scenario 1), a decision accuracy of 98.5% was achieved in multi-lane merging areas, demonstrating the system’s ability to effectively mitigate the challenges posed by occlusion and make timely, precise decisions in complex intersection settings. Although the decision accuracy slightly dropped to 96.9% in unprotected left-turn intersections, the system still maintained high reliability, confirming its capability to handle diverse and fluctuating road conditions. Furthermore, in the scenario where pedestrians suddenly entered the roadway (Emergency Scenario 2), the system reached an impressive decision accuracy of 98.7% in multi-lane merging zones, underscoring its rapid identification and response to unexpected events in environments with multiple dynamic factors. This performance reflects exceptional dynamic responsiveness in high-risk situations.

In the context of vehicle lane-changing violations (Emergency Scenario 3), the system’s decision-making accuracy surpassed 98% across all test scenarios, with the highest performance recorded in multi-lane merging areas (99.0%). This highlights the system’s precision in addressing prevalent traffic behaviors, swiftly detecting abnormal driving actions, and formulating optimized decisions. In the scenario where a preceding vehicle initiates an emergency brake (Emergency Scenario 4), decision accuracy fluctuated between 97.6% and 98.3%. Although slight performance dips were observed at unprotected left-turn intersections, the system consistently demonstrated efficient responsiveness across diverse complex road conditions, maintaining safety and stability. Across all scenarios, particularly in multi-lane merging areas and during unforeseen traffic events, the system exhibited robust stability and high efficiency in decision-making, significantly improving safety in complex intersection environments by making rapid and accurate decisions.

**Fig. 8:**
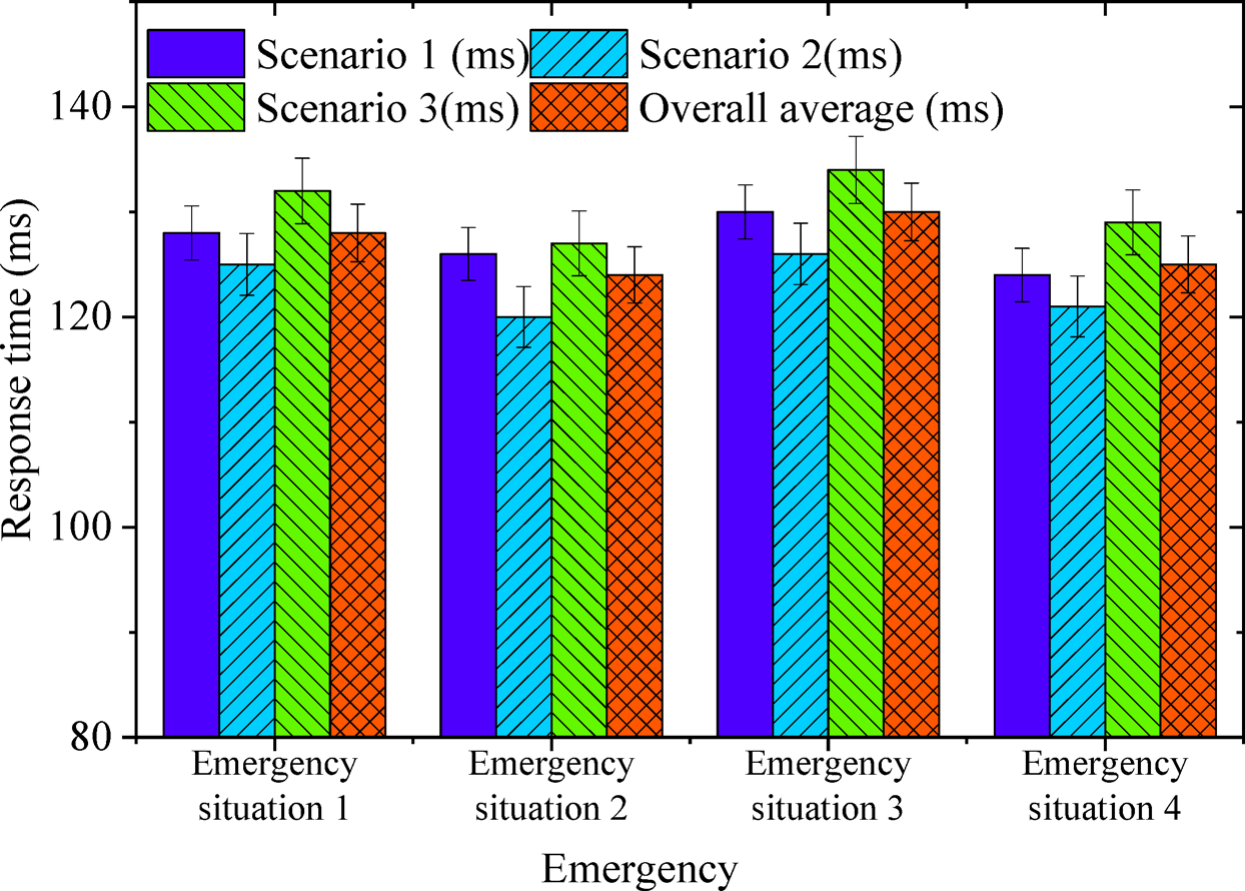
Response time under different emergency situations (milliseconds).

Figure 8 offers additional insights into response times (in milliseconds) across various emergency scenarios and urban complex intersection environments.

The system maintains consistently short response times across various complex scenarios, underscoring its strong real-time responsiveness in high-risk environments and its ability to execute rapid decision-making in multiple emergency situations. In Emergency Scenario 1 (obstacles), a slight increase in response time was observed in the multi-lane merging area (132ms), while overall response times remained around 128ms, demonstrating the system’s adaptability to dynamic environmental changes. At signal-controlled intersections and unprotected left-turn intersections, response times measured 128ms and 125ms, respectively, reflecting the system’s capacity to sustain stable reaction times across different traffic conditions and ensure prompt decision execution.

In Emergency Scenario 2 (vehicles in close proximity), the shortest response time (120ms) was recorded at unprotected left-turn intersections, whereas a marginally higher response time (127ms) was observed in the multi-lane merging area. The overall rapid response speed confirms the system’s ability to adjust efficiently within high-traffic, dynamically shifting environments. Emergency Scenario 3 (red light) resulted in a slight response time increase across different conditions, with the multi-lane merging area reaching 134ms. This suggests that heightened traffic interference in such environments may contribute to extended response times. However, with response times consistently maintained below 130ms, the system effectively manages emergency situations triggered by changes in traffic signals.

**Fig. 9:**
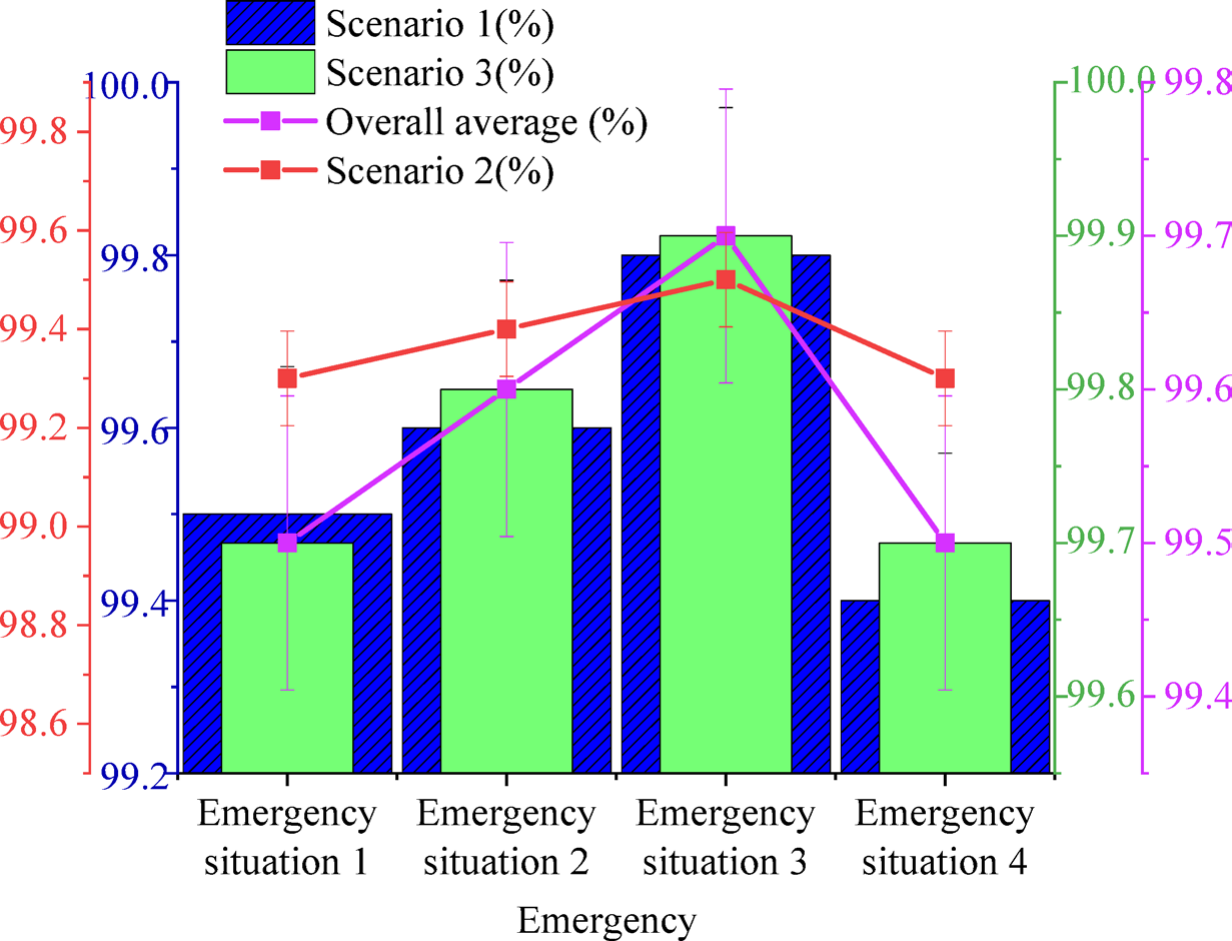
Hazard assessment accuracy under different emergency situations.

In Emergency Scenario 4 (sudden stop of the preceding vehicle), response time stability remained consistent across all tested conditions. The shortest response time was observed at the unprotected left-turn intersection (121ms), highlighting the system’s ability to rapidly adjust under dynamic intersection constraints. In contrast, a slightly higher response time of 129ms was recorded in the multi-lane merging area, though still well within an acceptable operational range. The overall average response time settled at 125ms, ensuring precise and timely decision execution in critical traffic situations. Across various complex urban traffic conditions, response times remained within a controlled range, reinforcing the system’s efficiency in high-density intersections. The capacity to maintain stable responsiveness, even under dynamically shifting conditions, underscores its adaptability in mitigating risks and optimizing emergency handling efficiency. Figure 9 further visualizes the system’s accuracy in risk assessment across diverse traffic complexities and emergency scenarios.

The results in Fig. 9 reveal consistently high accuracy across all evaluated scenarios, underscoring the system’s adaptability to dynamic and complex traffic conditions. In Emergency Scenario 1 (obstacle detection), risk assessment accuracy reaches 99.5% in urban streets, 99.3% on highways, and 99.7% in complex road segments. These findings highlight the system’s ability to efficiently detect and evaluate risks associated with obstacles, maintaining high precision across diverse road environments, including intricate intersections and high-speed corridors. Emergency Scenario 2 (short vehicle distance) further validates the system’s precision, with accuracy levels of 99.6% in urban streets, 99.4% on highways, and 99.8% in complex traffic conditions. These results demonstrate the system’s capability to assess proximity risks with high reliability, providing a robust foundation for real-time decision-making in high-risk scenarios.

In Emergency Scenario 3 (red light detection), the system achieves a peak accuracy of 99.9% in complex road segments, while maintaining similarly high performance in urban streets and highways. This level of precision ensures effective identification of red light violations, reinforcing timely risk assessments and contributing to optimized traffic management strategies. Emergency Scenario 4 (sudden stop of the preceding vehicle) presents another critical evaluation of risk assessment performance. Accuracy levels reach 99.4% in urban streets, 99.3% on highways, and 99.7% in complex environments, confirming the system’s ability to recognize and respond to sudden braking events with a high degree of reliability. Across all test conditions, the system demonstrates exceptional stability and accuracy in risk assessment. The ability to consistently identify and evaluate emergency traffic scenarios ensures efficient decision-making, enhancing both safety and operational effectiveness in rapidly changing traffic environments.

### Comparative analysis with existing methods

**Fig. 10:**
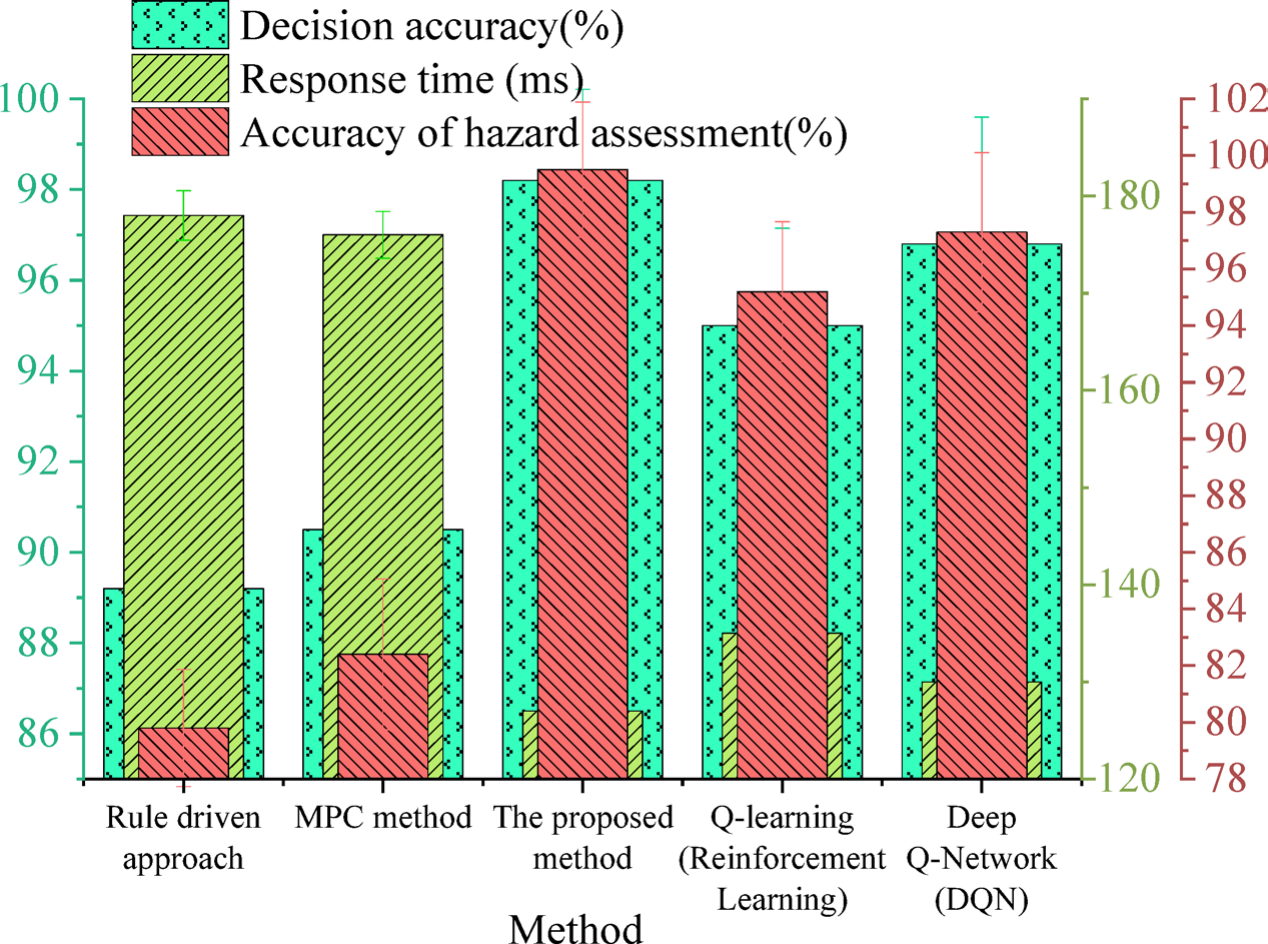
Performance comparison of different methods.

To systematically evaluate the effectiveness of the proposed hierarchical collaborative V2X decision optimization framework, a set of comparative experiments was designed. This includes current mainstream autonomous driving decision methods: rule-based decision models, MPC-based methods, and reinforcement learning approaches such as Q-learning and Deep Q-Networks (DQN). All comparison methods were run on the same CarSim + SUMO simulation platform and evaluated under uniform data input conditions to ensure fairness and consistency in the comparison. Specifically, the rule-based method employed an “If-Then” decision tree format, covering fundamental strategies like traffic light response, lane keeping, and obstacle avoidance. For the MPC method, a prediction horizon of 3 s was set, with 20 rolling optimizations performed within each control cycle. State variables include lateral and longitudinal velocity and position. Objective function weights were heuristically determined and optimized through cross-validation. The Q-learning model utilized an ε-greedy strategy for exploration, with an initial ε value of 0.9, linearly decaying to 0.05. The learning rate was set to 0.001, and training runs for 3,000 episodes. The DQN model used a two-layer fully connected network, with 128 neurons per layer and ReLU activation functions. It employed the Adam optimizer with a learning rate of 0.0005. The target network update frequency was set to 10 steps, and the experience replay buffer size was 50,000. All reinforcement learning models were trained using unified training data and environments, and their generalization capabilities were evaluated through 5-fold cross-validation. The performance metrics are presented in Fig. 10.

The data in Fig. 10 illustrate the substantial advantages of the proposed method in terms of decision accuracy, response time, and risk assessment precision. The framework achieves a decision accuracy of 98.2%, a response time of 127 milliseconds, and a risk assessment accuracy of 99.5%, demonstrating a marked improvement over conventional approaches. In contrast, the rule-based model records a decision accuracy of 89.2% and a response time of 178 milliseconds, while the MPC method achieves 90.5% decision accuracy with a response time of 176 milliseconds, indicating inferior performance across all key evaluation criteria. Reinforcement learning-based models exhibit notable enhancements over rule-based and MPC methods. The DQN achieves a decision accuracy of 96.8% with a response time of 130 milliseconds, outperforming traditional methods. However, this is still slightly lower than the proposed HRL-based V2X framework. Similarly, Q-learning, while yielding a decision accuracy of 95.0% and a response time of 135 milliseconds, surpasses conventional methods but does not reach the performance level of the proposed approach. These findings highlight the advantages of the proposed hierarchical collaborative V2X decision optimization framework in enhancing the decision-making efficiency of autonomous driving, particularly under high-risk and dynamic traffic conditions.

The fundamental reason for these performance differences lies in the HRL mechanism and the multi-agent collaborative structure introduced within the proposed framework. The HRL structure models high-level and low-level policies in a hierarchical manner, making the decision process more structured and flexible. This provides stronger generalization capabilities and real-time adjustment abilities, especially when handling high-risk, complex traffic scenarios. Concurrently, the multi-agent collaborative mechanism supports information sharing and policy cooperation among vehicle, roadside, and cloud agents. This achieves higher-level environmental modeling and task allocation, significantly enhancing the system’s dynamic adaptability and global optimality. Unlike DRL-based models, rule-based approaches rely on predefined decision rules, resulting in limited flexibility and adaptability. MPC methods, despite their optimization potential, demonstrate lower competitiveness when compared to reinforcement learning-based adaptive frameworks due to constraints in handling highly dynamic environments.

The integration of a cloud-based control platform further enhances the effectiveness of the V2X decision optimization framework by leveraging advanced data processing and analytical capabilities. Through real-time vehicle status monitoring and sensor data-driven dynamic decision optimization, the cloud platform significantly improves system adaptability and response efficiency. In high-risk road segments or traffic-intensive environments, real-time traffic information is utilized to refine decision strategies, thereby enhancing overall situational awareness and ensuring precise decision-making. Furthermore, the collaborative decision-making mechanism facilitated by the cloud platform effectively alleviates computational burdens on local processing units, optimizes decision accuracy, and reduces response latency, thereby reinforcing system reliability in complex traffic scenarios.

Unlike traditional methods that rely on static rules or short-term optimization, this framework leverages a cloud platform to integrate continuous learning with global optimization capabilities. The HRL model not only continually updates its policies from local experiences but also dynamically incorporates large-scale traffic information through the cloud’s cross-scenario learning ability. This enhances its generalization and real-time response capabilities across diverse scenarios. The cloud-based collaborative mechanism further amplifies HRL’s optimization efficiency in high-dimensional state spaces, ensuring decision robustness in complex and dynamic environments.

In summary, the proposed hierarchical collaborative V2X decision optimization framework, by introducing HRL, a multi-agent collaboration mechanism, and a cloud-based decision platform, outperforms traditional methods across several key performance indicators. It demonstrates greater practicality and safety in dynamic traffic scenarios, fully proving its potential and forward-looking value in autonomous driving systems.

## Conclusion

This research introduces a V2X-based hierarchical collaborative decision optimization framework. It integrates HRL with a multi-agent collaboration mechanism to achieve efficient linkage among vehicle, roadside, and cloud entities. This significantly enhances the decision-making efficiency and safety of autonomous driving systems in complex traffic scenarios. The HRL structure divides decision-making into two levels—policy planning and action execution—which improves the system’s adaptability and generalization capabilities. The multi-agent collaborative optimization further boosts the system’s response speed and global consistency during dynamic interactions. Additionally, the framework incorporates a CNN-based road segment risk classification model. This model achieves deep fusion of multi-source data and high-precision risk identification, providing prior safety information to support the decision-making process. Experimental validation demonstrates that this framework surpasses existing methods in key performance indicators such as decision accuracy, response time, and risk assessment precision. This effectively supports the intelligent and practical development of autonomous driving systems. Despite the demonstrated effectiveness of this approach, validation has been conducted primarily within simulation environments and through publicly available datasets. Although the results indicate significant improvements, assessing the framework under real-world driving conditions remains essential to determine its robustness and adaptability. The current hazard classification model relies heavily on historical data and sensory perception, limiting its responsiveness to abrupt environmental changes. Addressing these challenges requires future research to focus on real-world implementation and the construction of a large-scale autonomous driving dataset, ensuring comprehensive validation across diverse and complex road conditions.

## Electronic supplementary material

Below is the link to the electronic supplementary material.

Supplementary Material 1.

## Data Availability

Data is provided within the manuscript or supplementary information files.
